# Pregnane X receptor is associated with unfavorable survival and induces chemotherapeutic resistance by transcriptional activating multidrug resistance-related protein 3 in colorectal cancer

**DOI:** 10.1186/s12943-017-0641-8

**Published:** 2017-03-29

**Authors:** Yan Dong, Zhe Wang, Gan-feng Xie, Chong Li, Wen-wei Zuo, Gang Meng, Cheng-ping Xu, Jian-jun Li

**Affiliations:** 1 Department of Oncology, Southwest Hospital, Third Military Medical University, No. 29, Gaotanyan Street, Shapingba District, Chongqing, 400038 People’s Republic of China; 2 Department of Pathology, Southwest Hospital, Third Military Medical University, No. 29, Gaotanyan Street, Shapingba District, Chongqing, 400038 People’s Republic of China

**Keywords:** Colorectal cancer, Chemotherapy resistance, PXR, MRP3, Transcriptional regulation, Overall survival

## Abstract

**Background:**

Although chemotherapy represents a predominant anti-cancer therapeutic modality, drug treatment efficacy is often limited due to the development of resistant tumor cells. The pregnane X receptor (PXR) affects chemotherapeutic effects by regulating targets involved in drug metabolism and transportation, but the regulatory mechanism is poorly understood.

**Methods:**

Oxaliplatin (L-OHP) content in tumor cells was analyzed by mass cytometry. The roles of PXR on cancer cell proliferation, apoptosis and tumor growth with L-OHP-treated were investigated by MTS, colony formation, flow cytometry and xenograft tumor assays. Luciferase reporter, Chromatin-immunoprecipitation and Site-directed mutagenesis were evaluated the mechanisms. The PXR and multidrug resistance-related protein 3 (MRP3) expressions were examined by western blot, RT-PCR or immunohistochemistry of TMA. Kaplan-Meier and Cox regression were adopted to analyze the prognostic value of PXR in colorectal cancer (CRC).

**Results:**

PXR over-expression significantly increased oxaliplatin (L-OHP) transport capacity with a reduction of its content and repressed the effects of L-OHP on tumour cell proliferation and apoptosis. Conversely, PXR knockdown augments L-OHP-mediated cellular proliferation and apoptosis. Moreover, PXR significantly reduced the therapeutic effects of L-OHP on tumor growth in nude mice. Further studies indicated a positive correlation between PXR and MRP3 expression and this finding was confirmed in two independent cohorts. Significantly increased MRP3 expression was also found in PXR over-expressing cell lines. Mechanistically, PXR could directly bind to the MRP3 promoter, activating its transcription. The PXR binding sites were determined to be at -796 to -782bp (CTGAAGCAGAGGGAA) and the key binding sites were the “**AGGGA**” (-787 to -783bp) on the MRP3 promoter. Accordingly, blockade of MRP3 diminishes the effects on drug resistance of PXR. In addition, PXR expression is significantly associated with poor overall survival and represents an unfavorable and independent factor for male or stage I + II CRC patient prognosis.

**Conclusions:**

PXR is a potential biomarker for predicting outcome and activates MRP3 transcription by directly binding to its promoter resulting in an increased L-OHP efflux capacity, and resistance to L-OHP or platinum drugs in CRC. Our work reveals a novel and unique mechanism of drug resistance in CRC.

**Electronic supplementary material:**

The online version of this article (doi:10.1186/s12943-017-0641-8) contains supplementary material, which is available to authorized users.

## Background

Colorectal cancer (CRC), a malignancy of the gastrointestinal tract, is the third most common tumor worldwide [1]. CRC patient outcomes have significantly improved due to the use of novel treatment modalities and complex treatment strategies, including optimized surgery, adjuvant chemotherapy and novel targeted biological agents. At an early stage, CRC is potentially curable by surgery followed by chemotherapy [2]. However, approximately 25% of CRC patients present with metastatic disease at diagnosis and are unlikely to undergo curative surgical resection, and many patients with metastatic disease will relapse after potentially curative resections [3, 4]. For these patients, systemic chemotherapy is most often the treatment of choice for increasing survival and improving quality of life [5, 6]. Thus, chemotherapy is one of the most important treatments for malignancies in humans [7]. Previous studies have revealed that the response rate after initial chemotherapy treatment is approximately 50% for CRC patients [8, 9]. However, only 10% of CRC patients respond to secondary treatments [8]. These data suggest that there is a high degree of resistance to current chemotherapies.

The pregnane X receptor (PXR/NR1I2) is a ligand-activated transcription factor belonging to the nuclear hormone receptor (NR) superfamily [10]. PXR is expressed in many normal human tissues, including ovary, small intestine, colon, liver, breast, heart, and prostate [11]. Furthermore, increased PXR expression has been reported in various tumors, such as breast, ovarian, colon and endometrial cancers [12–17]. Further studies have shown that PXR plays an important role in the development of resistance to certain chemotherapeutic cancer treatments [18–22]. PXR activates many drug-metabolizing enzymes and drug transporters, resulting in altered drug clearance [23]. Our previous study showed that the mRNA expression of MRP3 significantly correlates with PXR expression in CRCs, suggesting that MRP3 might be involved in PXR-mediated chemotherapeutic resistance [16]. However, the specific role and precise molecular mechanism of PXR- and MRP3-mediated chemotherapeutic resistance remain unclear.

In the present study, we demonstrated that PXR significantly reduced the amount of oxaliplatin (L-OHP) in tumor cells, and prevented L-OHP-mediated inhibition of cellular proliferation and induction of apoptosis. Our nude mouse model showed that PXR reduced the efficacy of L-OHP treatment on tumor growth. Further analysis revealed a positive correlation between MRP3 and PXR expression (*P* = 0.0023), which was confirmed in two independent cohorts from The Cancer Genome Atlas (TCGA) database. We also detected increased MRP3 expression in PXR over-expressing cell lines. Moreover, luciferase reporter, ChIP and site-directed mutagenesis assays demonstrated that PXR could directly bind to the MRP3 promoter, and that the key binding site were located at -796 to -782bp on the MRP3 promoter. Further investigations showed that PXR expression was associated with poor prognosis and was an independent prognostic factor in CRC. Our data reveal that PXR can transcriptionally activate MRP3 expression by directly binding to its promoter, which increases drug efflux and results in CRC resistance to L-OHP or platinum drugs.

## Methods

### Patient samples

A total of 93 colorectal cancerous and 87 corresponding noncancerous tissues were obtained from the Southwest Hospital in Chongqing, China. This study was approved by the ethics committee of Southwest Hospital. Informed consent was obtained from all recruited patients.

### Tissue microarray generation and immunohistochemical analysis

Noncancerous adjacent tissues were compared with normal tissue, stained with haematoxylin-eosin and reviewed by at least 2 pathologists. The tissue microarray (TMA) containing tissues from 93 tumors with 87 pairs of tumor and matched peritumoural tissues, was constructed (in collaboration with Shanghai Biochip Company Ltd, Shanghai, China) as previously described [24].

Immunohistochemistry (IHC) was performed using PXR and MRP3 antibodies (both 1:200; both from Santa Cruz Biotechnology; sc-48403 and sc-5774). Tumor cell staining was considered positive when immunoreactivity was greater than or equal to 10%. Positive staining was divided into 5 categories, and staining intensity was graded according to 4 levels as previously described [25]. The expression levels were defined by the sum of positive staining and intensity.

### The construction and transfection of expression and siRNA vectors for PXR

Expression and siRNA vectors for PXR were constructed and transfected as previously described [26]. The stably PXR-transfected cells were selected using G418 (Calbiochem, La Jolla, CA, USA). Cell clones were obtained using the cylinder method.

### RT-PCR and western blotting (WB) analysis

The mRNA expression analysis was performed using RT-PCR. Series PCR assays with different cycle numbers were performed to determine the linear phase of amplification. Based on the pilot experiments, the appropriate cycles were chosen. The primers are listed in Additional file 1: Table S1.

Sixty micrograms of protein was resolved by 10–15% sodium dodecyl sulphate- polyacrylamide gel electrophoresis and transferred onto polyvinylidene difluoride membranes (Millipore Corporation, Bedford, MA, USA). After blocking, the membranes were incubated with primary antibodies overnight at 4 °C. The proteins were detected by chemiluminescence (Pierce, Rockford, IL, USA) after incubation with secondary antibodies. The probed membranes were stripped and incubated with β-actin monoclonal antibody (1:1200; Sigma). The primary antibodies used were PXR mouse monoclonal antibody (1:1000, Santa Cruz Biotechnology, sc-48403) and MRP3 goat polyclonal antibody (1:800; Santa Cruz Biotechnology, sc-5774). The secondary antibodies used were horseradish peroxidase-conjugated anti-mouse and anti-goat antibodies (both 1:2000, Jackson ImmunoResearch Laboratories, Inc., West Grove, PA, USA).

### Oxaliplatin drug treatment

For L-OHP content measurement, tumor cells were treated for 2 h at a concentration of 20mmol/L L-OHP (Hengrui Medicine, Lianyungang, Jiangsu Province, China); For the functional experiments in vitro, tumor cells were exposed for 24 h to 20 mmol/L L-OHP. The culture medium was replaced on a daily. For the tumor formation in vivo, the nude mice were injected once every 3 days with 5 mg of L-OHP (dissolved in normal saline)/kg body weight. Controls were treated with the same volume of normal saline.

### Measurement of L-OHP content in tumour cells by mass cytometry

The transfected, L-OHP-treated tumor cells were analyzed using a CyTOF1 mass cytometer (DVS Sciences, Richmond Hill, Ontario, Canada). The instrument settings and initial post-processing parameters were set as previously described [27–29]. Cells were measured at approximately 600 cells per second. Noise reduction was activated, and cell extraction parameters were as follows: cell length range was set from 15- to- 65 pushes, and the lower convolution threshold was set to 15.

### Colony-formation and MTS assay

HCT116 and LOVO cells were plated in 12-well plates at 3 × 10^5^ cells per well. After culturing for 24 h, the cells were transfected with PXR or vector plasmids. After 48h of transfection, the cells were collected, diluted 1:5, plated in 12-well plates and selected with 0.8mg/ml of G418 for 14 days to establish stable clones and for subsequent treatment with L-OHP. Surviving colonies were stained by using Giemsa’s azur eosin methylene blue solution (Merck, Darmstadt, Germany) and counted. These experiments were performed in triplicate.

HCT116 and LOVO cells were plated in 96-well plates at 3.5 × 10^3^ cells per well, transfected with PXR or empty vector plasmids, and treated with L-OHP. For knockdown, SW480 cells were plated in 96-well plates at 3 × 10^3^ cells per well, transfected with PXR siRNA or negative control plasmids, and treated with L-OHP. Cell proliferation was evaluated using the Cell Proliferation Reagent MTS (CellTiter 96® Aqueous One Solution Cell Proliferation Assay, Promega) on days 1, 2, and 3. The assay was carried out in triplicate in three independent experiments.

### Flow cytometry assay

PXR or empty vector-transfected cells (3.5 × 10^5^ cells per well) were harvested at 48 h post-transfection and L-OHP treatment, and fixed in 70% ethanol overnight at 4 °C. The cells were stained with propidium iodide (BD Pharmingen, San Jose, CA, USA). Fifty thousand cells were sorted by FACSCalibur System (BD Biosciences, Franklin Lakes, NJ, USA), and cell cycle profiles were analysed using the ModFit software (Verity Software House, Topsham, ME, USA). Apoptosis was also assessed by Annexin V-APC/7-amino-actinomycin D staining (KeyGEN, Nanjing, China). Briefly, cells were harvested, washed with phosphate-buffered saline, resuspended in 500 μl of binding buffer, mixed with 5 μl of Annexin V-APC and 5μl of 7-ADD, incubated for 5–15 min in the dark. Fifty thousand cells were analyzed using a FACSCalibur cytometer. The Annexin V-positive cells were considered apoptotic cells and analysed using the ModFit software. The assays were carried out in triplicate in three experiments.

### In vivo tumourigenicity

To assess the effects of PXR expression and L-OHP treatment on tumour formation in vivo, the PXR- or empty vector-expression stable HCT116 cells were injected subcutaneously into the right flanks of 6-week-old male Balb/c nude mice that were subsequently subjected to L-OHP treatment. Tumor volume was calculated as 0.5236 L1 (L2)^2^ [30]. The developing tumors were observed during the following 5 weeks, after which the mice were sacrificed. All mouse experiments were approved by the Institutional Animal Care and Use Committee of Third Military Medical University, China.

### Site-directed mutagenesis of binding site assay

The site-directed mutagenesis assays were performed as previous study (26). The binding sites in MRP3 promoter constructs were mutated or deleted using a Quik- Change Lightning Multi Site-Directed Mutagenesis Kit (Stratagene, La Jolla, CA, USA) according to the instructions, and the mutations or deletions were confirmed by direct DNA sequencing.

### Luciferase reporter and chromatin-immunoprecipitation (ChIP) assay

Cells were seeded in 24-well plates at 3.5 × 10^4^ cells per well. After an overnight incubation, the cells were transfected with a DNA mixture including pGL3-MRP3 promoter-luciferase, pIRES2-EGFP-PXR or vector, as well as the internal pRL-TK plasmids. Luciferase activities were measured at 36 h post-transfection using a dual-luciferase reporter kit (Promega). Each experiment was performed in triplicate and repeated three times.

Chromatin-immunoprecipitation (ChIP) analysis was performed using a ChIP Assay Kit (Cell Signaling Technology) according to the manufacturer’s protocols. The immunoprecipitated and input DNA were used as templates for RT-PCR analysis, and the primers are listed in Additional file 1: Table S1.

### Statistical analyses

Statistical analyses were performed using SPSS 13.0 software (SPSS, Inc., Chicago, IL, USA). The results are expressed as the mean ± standard deviation (s.d.). The data were analysed by Student’s *t* and chi-square (2-sided) tests. Patient clinical and pathological characteristics were compared by Pearson *χ* ^2^ test. Overall survival (OS) was calculated according to Kaplan-Meier and Cox regression. The p values less than 0.05 were considered significant.

## Results

### PXR decreases oxaliplatin (L-OHP) levels in tumor cells

**Fig. 1:**
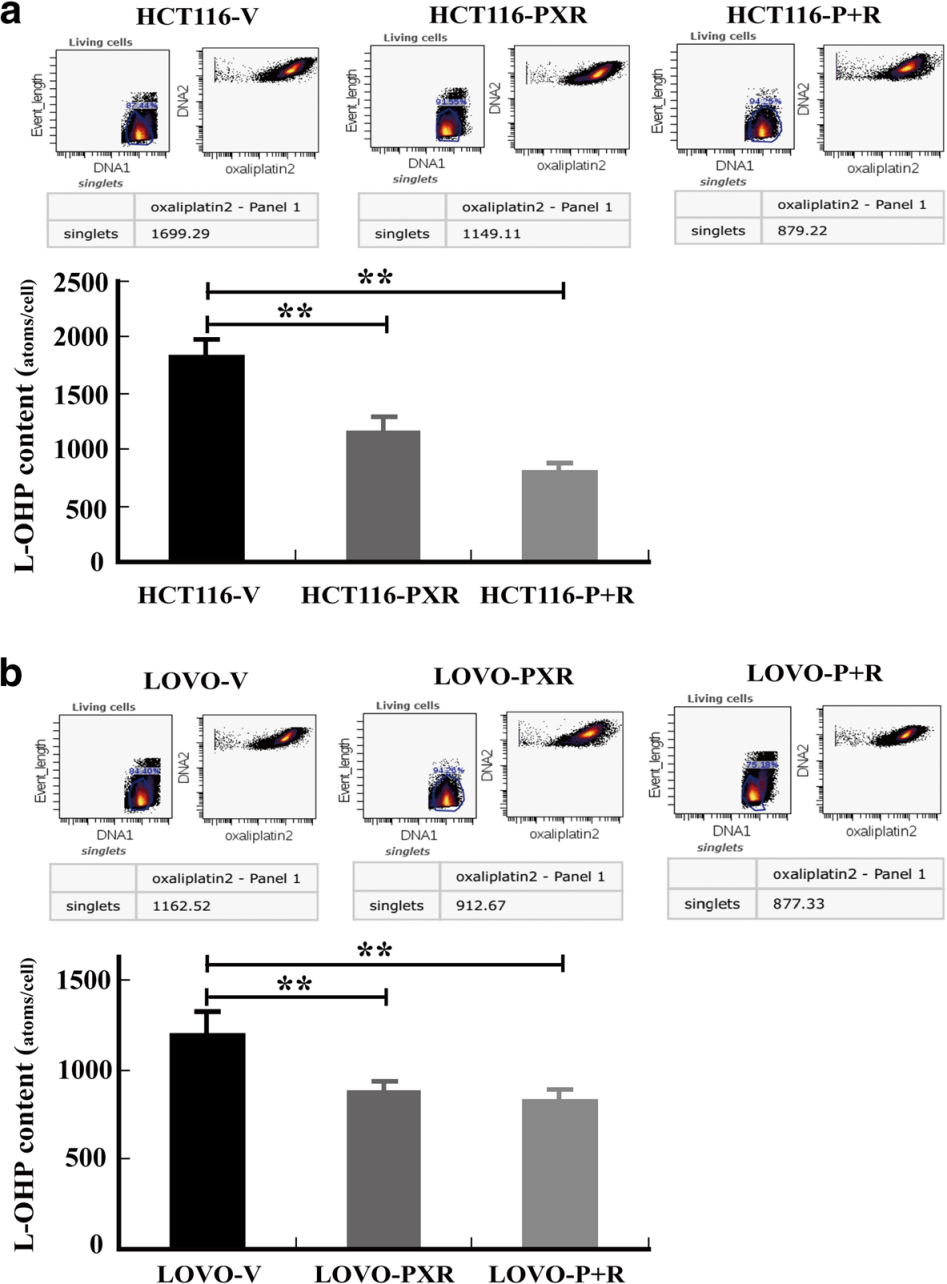
PXR over-expression decreases L-OHP levels in tumor cells. The transport and uptake of L-OHP in tumor cells were assessed by mass cytometry. L-OHP content in PXR or PXR + RXRA stably transfected tumor cells was reduced compared with that in empty vector stably transfected tumor cells on average in both HCT116 (**a**) and LOVO (**b**) cells. Tumour cells were treated for 2 h with 20 mmol/L oxaliplatin. P + R, PXR + RXRA. The unit shown is Pt atoms/cell

To determine the role of PXR in L-OHP-treated tumor cells, L-OHP transport and uptake were assessed by mass cytometry in tumor cells transfected with PXR or empty vector, as well as in those transfected with PXR + RXRA, which generally forms a heterodimer that transcriptionally activates target genes. Our results revealed that the L-OHP content in tumor cells stably transfected with PXR or PXR + RXRA was on average significantly lower than that in tumor cells transfected with empty vector in both HCT116 and LOVO cells (Fig. 1a, b). These results showed that PXR notably increased the L-OHP efflux capacity of tumor cells, thereby reducing intracellular L-OHP content.

### PXR prevents L-OHP-mediated inhibition of cancer cell proliferation and apoptosis

**Fig. 2:**
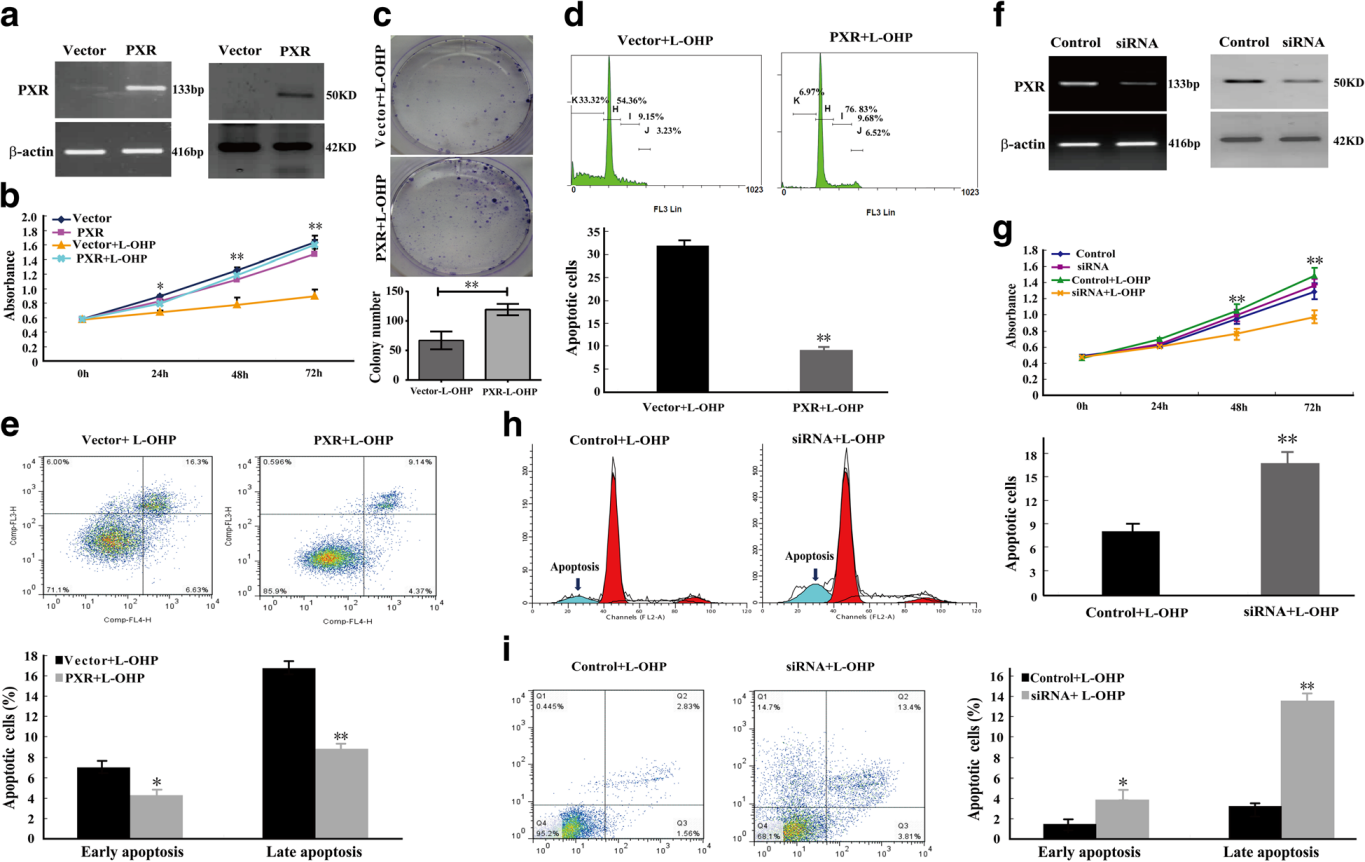
PXR prevents L-OHP-mediated tumor cell growth inhibition and induction of apoptosis. **a** PXR and empty vector control transfectants were identified by RT–PCR and WB in HCT116. Abundant PXR was detected in PXR transfectants but not in empty vector transfectants. β-actin was used as an internal control. **b** MTS assays were used to examine the effects of PXR on L-OHP-mediated suppression of tumor cell proliferation. Cell viability was evaluated in triplicate. *P < 0.05; **P < 0.01. **c** The effects of PXR on L-OHP-mediated cell growth were further confirmed using colony-formation assays. The colonies were counted. The error bars indicate the s.d. (*n* = 3), **P < 0.01. **d** The effects of PXR on L-OHP-induced cellular apoptosis were detected by propidium iodide flow cytometric assessment of the sub-G1 fraction. The error bars indicate the s.d. (*n* = 3). **e** The effects of PXR on L-OHP-induced cellular apoptosis were detected by Annexin V-APC/7-amino-actinomycin D flow cytometry. The error bars indicate the s.d. (*n* = 3). **f** PXR knockdown was confirmed by RT–PCR and WB in SW480 cells. β-actin was used as an internal control. **g** An MTS assay was performed to assess cell proliferation after PXR knockdown and treatment with L-OHP. Cell viability was evaluated in triplicate. *P < 0.05; **P < 0.01. **h** The effects of PXR knockdown on L-OHP-induced cellular apoptosis were detected by propidium iodide flow cytometric assessment of the sub-G1 fraction. The error bars indicate the s.d. (*n* = 3). **i** Effect of PXR knockdown on L-OHP-induced cell apoptosis was detected by Annexin V-APC/7-amino-actinomycin D flow cytometry. The error bars indicate the s.d. (*n* = 3)

To explore the functional roles of reduced L-OHP content, we performed cell proliferation assays in L-OHP-treated PXR over-expressing cells. The MTS data and colony-formation assays indicated that PXR prevented the L-OHP-mediated suppression of tumor cell proliferation (Fig. 2a-c, Additional file 1: Table S2). To exclude the possible direct role of PXR over-expression, the data of MTS assay presented also include groups without L-OHP treatment (Fig. 2b). To further examine the effects of PXR over-expression on the L-OHP-mediated inhibition of cellular proliferation, we measured the percentage of sub-G1 phase cells and also performed Annexin V-APC/7-amino-actinomycin D staining followed by flow cytometric analysis. The PXR-transfected tumor cells exhibited a significantly reduced percentage of sub-G1/apoptotic cells as compared with empty vector-transfected cells that were treated with L-OHP (Fig. 2d). The double staining analysis revealed that PXR over-expression decreased the percentage of early- and late-stage apoptotic cells after L-OHP treatment (Fig. 2e). These results demonstrated that PXR over-expression promoted tumor cell proliferation and inhibited tumor cell apoptosis during L-OHP treatment.

To further demonstrate that PXR expression can influence tumor cell proliferation and apoptosis during L-OHP treatment, we constructed a PXR-knockdown SW480 cell model (Fig. 2f). Cell proliferation and flow cytometry assays revealed that PXR knockdown inhibited tumor cell proliferation and promoted apoptosis after L-OHP treatment (Fig. 2g-i). The data of MTS assay presented also include groups without L-OHP treatment (Fig. 2g). These results further demonstrated that PXR promoted tumor cell proliferation and inhibited L-OHP-induced tumor cell apoptosis.

### PXR reduces the curative effect of L-OHP on tumor growth in nude mice

**Fig. 3:**
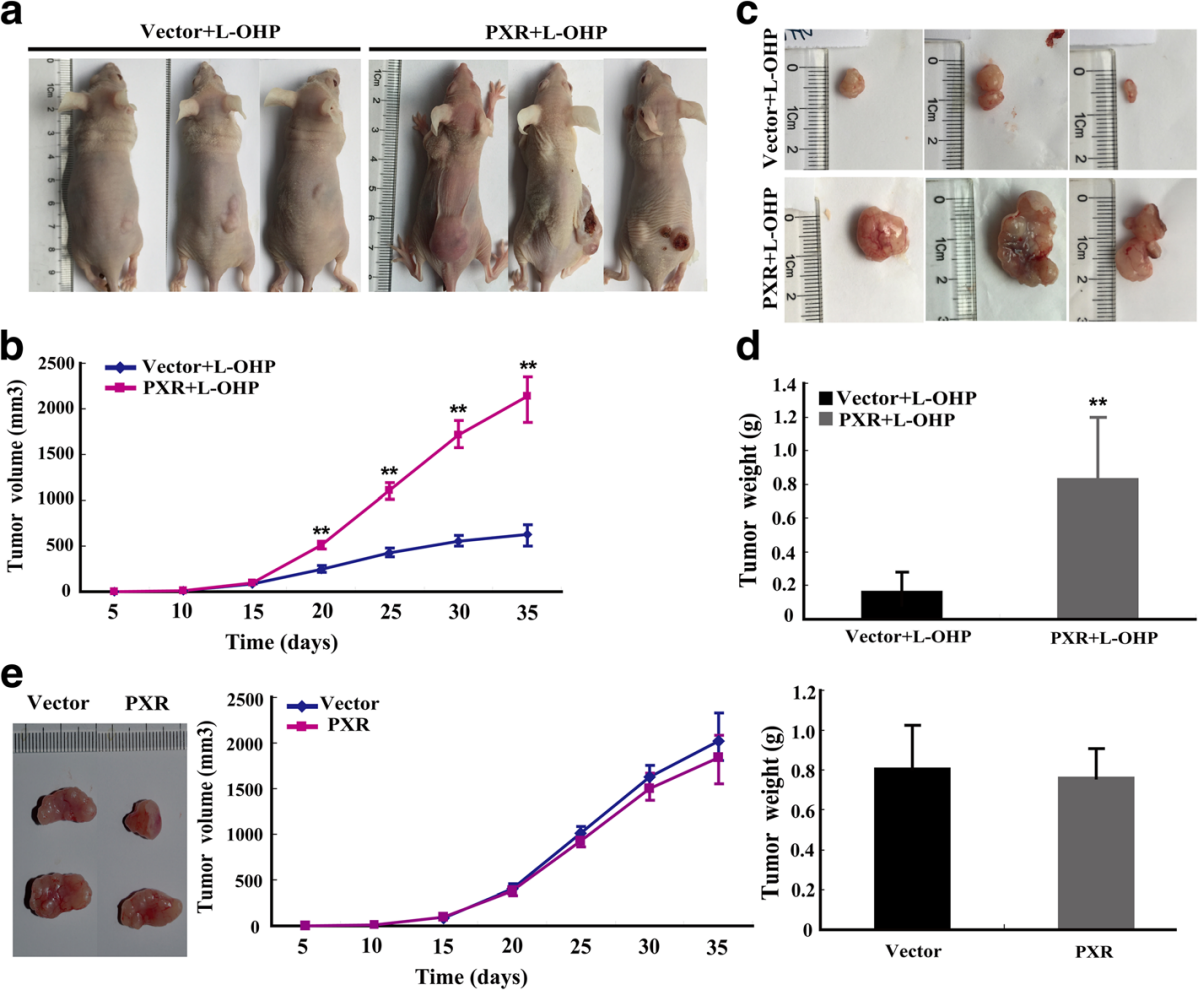
PXR reduces the curative effect of L-OHP on tumor growth in nude mice. **a**, **b** The tumor growth curve of PXR-expressing cells was compared with vector-expressing cells after L-OHP treatment. Tumor growth was assessed in nude mice that were subcutaneously injected in the right flank with 5.0 × 10^6^ stable transfectants. Points represent the mean tumor volumes of three independent experiments (*n* = 3). **c**, **d** Tumor weights from the PXR and vector groups were measured. The results were obtained from three independent experiments. *P < 0.05; **P < 0.01. The error bars indicate the s.d. (*n* = 3). **e** The tumor growth curve and tumor weights of PXR-expressing cells were compared with vector-expressing cells. The results were obtained from three independent experiments. The error bars indicate the s.d. (*n* = 3)

Our experiments demonstrated that PXR can promote tumor cell proliferation and inhibit tumor cell apoptosis during L-OHP treatment in vitro. To test this conclusion in vivo, we assessed tumourigenicity in a nude mouse model of stable PXR-expressing cells and L-OHP treatment. Tumor volumes were significantly larger in L-OHP-treated mice injected with PXR-transfected cells compared with those injected with empty vector-transfected cells (Fig. 3a, b). PXR over-expression resulted in an increase of the mean weight of tumors collected at 5 weeks after cell inoculation (Fig. 3c, d). To exclude the possible direct role of PXR, the data of tumor volumes and weights from nude mice were also presented without L-OHP treatment (Fig. 3e). Collectively, these data indicated that PXR significantly reduced the curative effects of L-OHP on tumor growth.

### PXR positively correlates with MRP3 expression in CRC

**Fig. 4:**
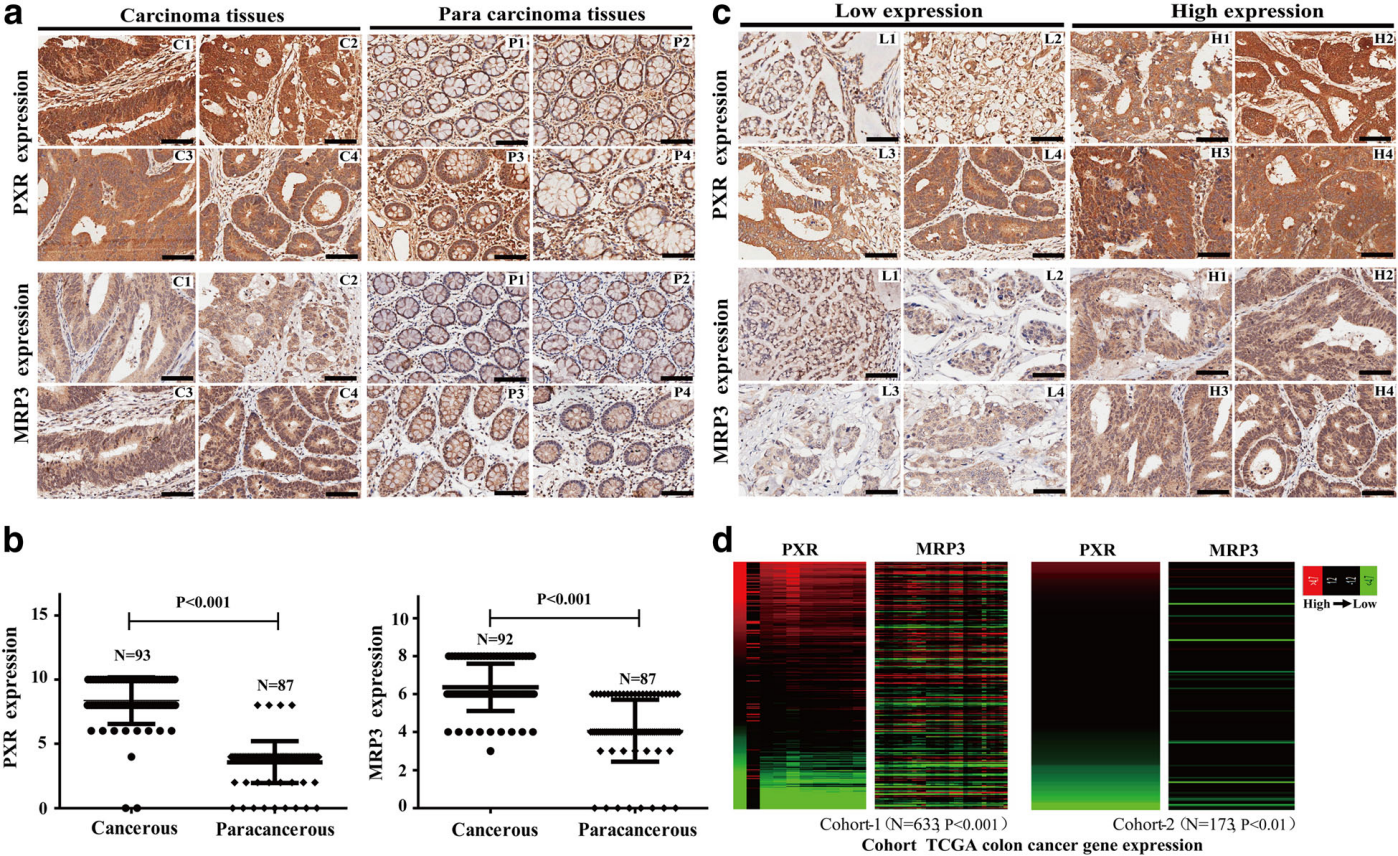
PXR positively correlates with MRP3 expression in colorectal cancer. **a** Immunohistochemical analysis of PXR and MRP3 expression was performed on cancerous and adjacent noncancerous samples. Low levels of PXR and MRP3 expression were observed in noncancerous tissues compared with cancerous tissues. Scale bars represent 50 μm. Representative samples were shown. **b** PXR and MRP3 expression levels were analyzed in cancerous and adjacent noncancerous tissues. PXR and MRP3 expression was higher in cancerous tissues than in adjacent noncancerous tissues. The error bars indicate the s.d. **c** A significant positive correlation between PXR and MRP3 expression was detected in colorectal cancer. The scale bars represent 50 μm. Representative samples were shown. **d** A significant positive correlation between PXR and MRP3 expression was also observed in two other independent colorectal cancer cohorts from the TCGA database. Heat map was generated to compare PXR and MRP3 expression in the TCGA colorectal cancer gene expression dataset (https://genome-cancer.soe.ucsc.edu/proj/site/xena/heatmap/)

To investigate the protein levels of PXR and MRP3 expression in human CRCs, we performed IHC for PXR and MRP3 in a TMA containing 93 cancer and 87 para-carcinoma colorectal tissues. PXR protein levels were increased in most cancer tissues compared with para-carcinoma tissues, and the mean expression of PXR in cancer tissues (8.366) was significantly increased compared with para-carcinoma tissues (3.586) (*P* < 0.001) (Fig. 4a, b). MRP3 protein levels were also increased in most cancers compared with para-carcinoma tissues, and the mean expression of MRP3 in the cancer tissues (6.361) was significantly increased compared with that of the para-carcinoma tissues (4.083) (*P* < 0.001) (Fig. 4a, b). These results suggested that PXR and MRP3 were frequently over-expressed in tumor tissues compared with in adjacent tissues.

To further evaluate whether PXR correlates with MRP3 expression, we assessed the relationship between MRP3 and PXR protein expression. Statistical analysis revealed a significant positive correlation between PXR and MRP3 protein expression in CRC (Fig. 4c, *P* = 0.0023), a result that was further confirmed in two independent cohorts from TCGA database at mRNA level (Fig. 4d). These results indicated that PXR might increase platinum drug metabolism, causing resistance to platinum drugs by up-regulating MRP3 gene expression.

### PXR activates MRP3 transcription by directly binding to its promoter

**Fig. 5:**
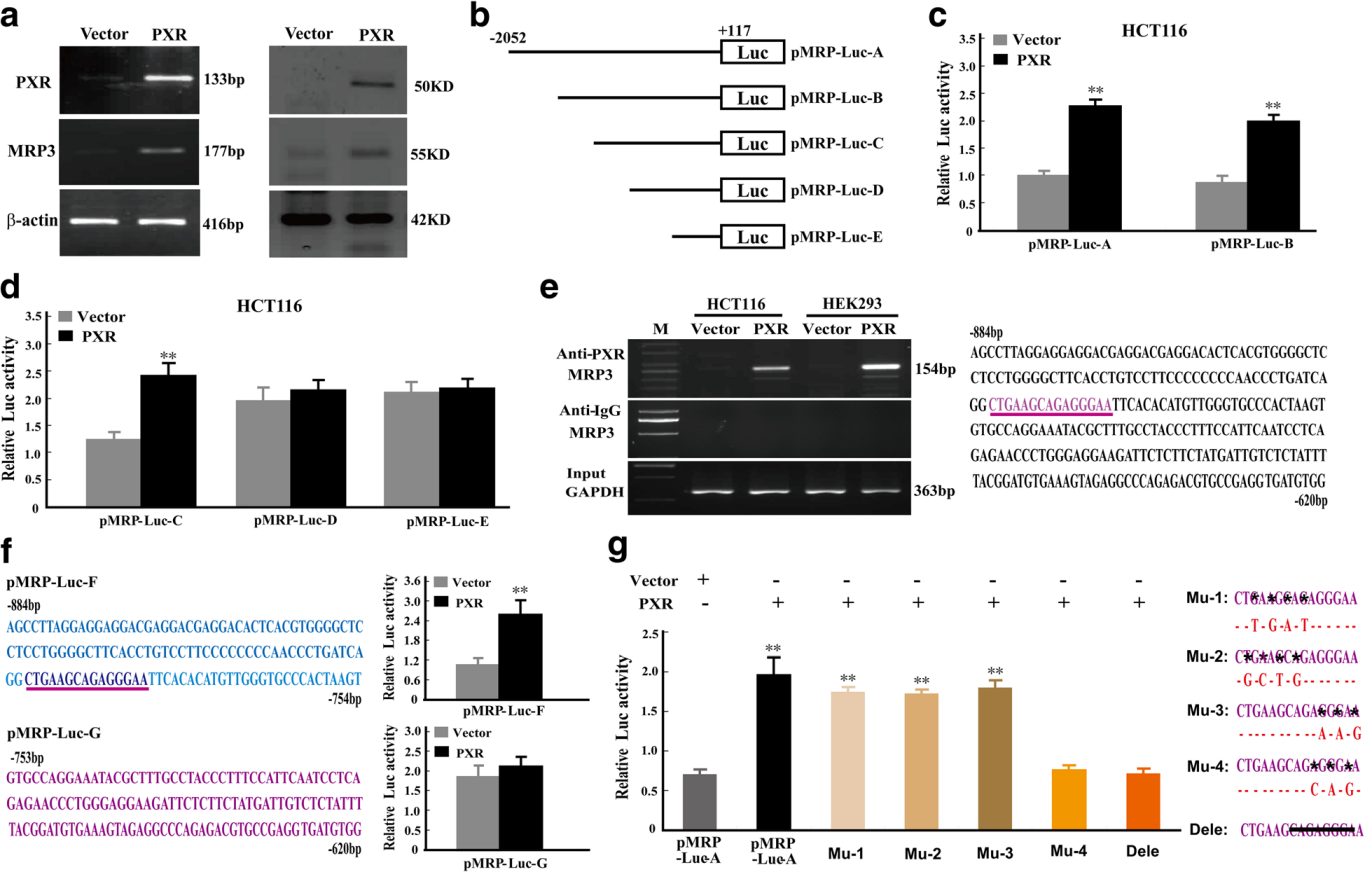
PXR activates MRP3 transcription by directly binding to its promoter. **a** Ectopic expression of PXR significantly enhances the endogenous expression of MRP3. PXR and MRP3 expressions were examined by RT-PCR and WB, and β-actin was used as an internal control. **b** Different regions of the MRP3 promoter were cloned into a luciferase reporter construct. **c**, **d** PXR targets MRP3 directly, and the key PXR binding region is located between -884 to -620bp of the MRP3 promoter as determined by luciferase reporter assays. The results were normalized to internal controls from three experiments. **e** ChIP-PCR was performed to identify MRP3 as a PXR target. Jaspar and ConSite predicted the PXR binding sites as located between -796 to -782bp of the MRP3 promoter. **f** The key PXR binding region is located between -884 to -754bp of the MRP3 promoter determined by luciferase reporter assays. The results were normalized to internal controls from three experiments. **P < 0.01. **g** The effects of PXR on wild-type and mutated/deleted MRP3 promoter (between -796 to -782bp) activity. Error bars indicate s.d. (*n* = 3), *, mutated sites; **-**, deleted sites. **P < 0.01

To elucidate the molecular mechanism underlying the PXR-mediated regulation of MRP3, gene expression profiling was performed using stable transfectants. Our data revealed that MRP3 expression increased with the increase of PXR expression (Fig. 5a). As PXR is a transcription factor, we first analyzed whether it could directly regulate MRP3 at the transcriptional level. To test this hypothesis, we performed a luciferase reporter assay, which revealed that PXR over-expression significantly enhanced the activity of the MRP3 promoter (-2052 to +117bp), suggesting that PXR regulates MRP3 by directly binding to its promoter. To identify the PXR-binding site(s) within the MRP3 promoter, different regions of the MRP3 promoter (pMRP-Luc-B- ~ E) were analyzed using luciferase reporter assays. Our results showed that the key region required for PXR binding was located between -884 to -620bp of the MRP3 promoter (Fig. 5b-d). This result was confirmed by a ChIP-PCR assay, and aided by the binding profile database of Jaspar and ConSite, we determined that the crucial binding region resides within -796 to -782bp (CTG AAGCAGAGGGAA) of the MRP3 promoter (Fig. 5e). To confirm the binding sites in the “CTGAAGCAGAGGGAA” region of the MRP3 promoter, two different regions of MRP3 promoter between -884 and -620bp (pMRP-Luc-F and G) were then analyzed using luciferase reporter assays. The data revealed that the region containing “CTGAAGCAGAGGGAA” region was required for PXR binding (Fig. 5f). To further investigate the key binding sites, constructs with site-directed mutagenesis of binding sites were generated. When the PXR-binding sites, ---------**AGGGA**-(-787 to -783bp), in MRP3 promoter were mutated or deleted, the activation was lost (Fig. 5g). These findings demonstrated that MRP3 was a direct PXR target gene in CRC.

### MRP3 is a key player in PXR mediated drug resistance

**Fig. 6:**
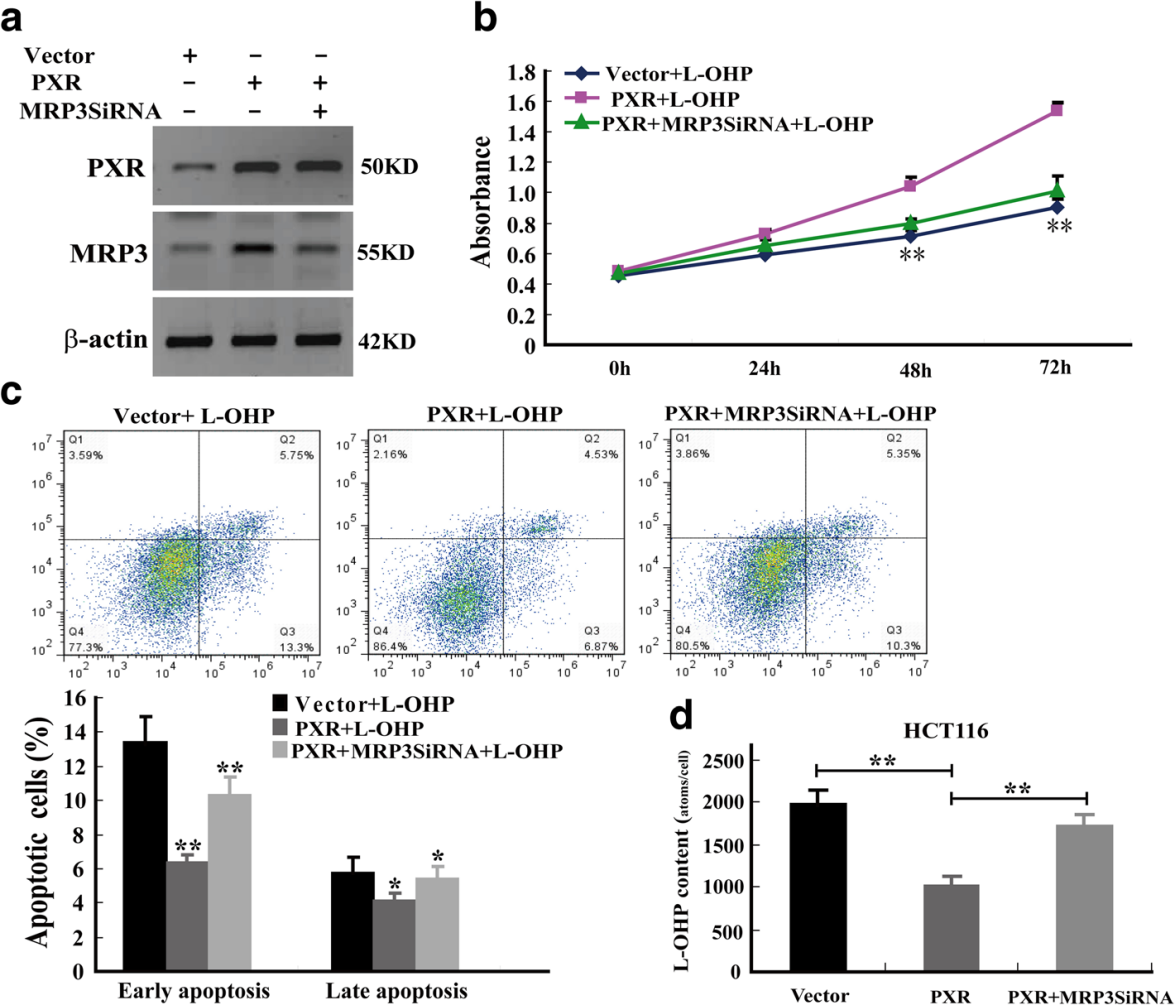
MRP3 is a functionally target gene of PXR in drug resistance. **a** The expression of PXR and MRP3 was analyzed by WB at 48h after transfecting with MRP3 small interfering RNA (siRNA). The MRP3 siRNA was purchased from Santa Cruz Biotechnology. **b** Analysis of the effects of L-OHP on cancer cell proliferation when different PXR and MRP3 expression. Cell numbers were counted every day after treatment for 4 days. **c** Analysis of the effects of L-OHP on cancer cell apoptosis when different PXR and MRP3 expression. **d** The transport and uptake of L-OHP in tumour cells with different PXR and MRP3 expression were assessed by mass cytometry

To define whether MRP3 was required for PXR mediated drug resistance in CRC, we performed a rescue experiment by knocking down MRP3 in PXR over-expressed cells followed by testing cellular proliferation, apoptosis and intracellular L-OHP content. The results of rescue experiment demonstrated that blockade of MRP3 by small interfering RNA (siRNA) significantly diminished the effect of PXR over-expression on cancer cell proliferation, apoptosis and intracellular L-OHP content (Fig. 6a–d). These data indicate that the contribution of MRP3 is very important for PXR mediated drug resistance in CRC.

### PXR expression is significantly associated with poor overall survival in < h2 > CRC patients

To determine the importance of PXR in CRC, the clinical and prognostic significance of PXR expression were analyzed. Based on the quantified positive staining of tumor cells, PXR expression was classified into two groups around the median score: as high (>median) and low (≤median). After investigating the association between PXR expression and the clinicopathological features of CRC patients, PXR expression was significantly correlated with patient age (*P* = 0.011), but not with sex, lymph node status, histological grade, tumor size and clinical stage (Additional file 1: Table S3 and Additional file 2).

**Fig. 7:**
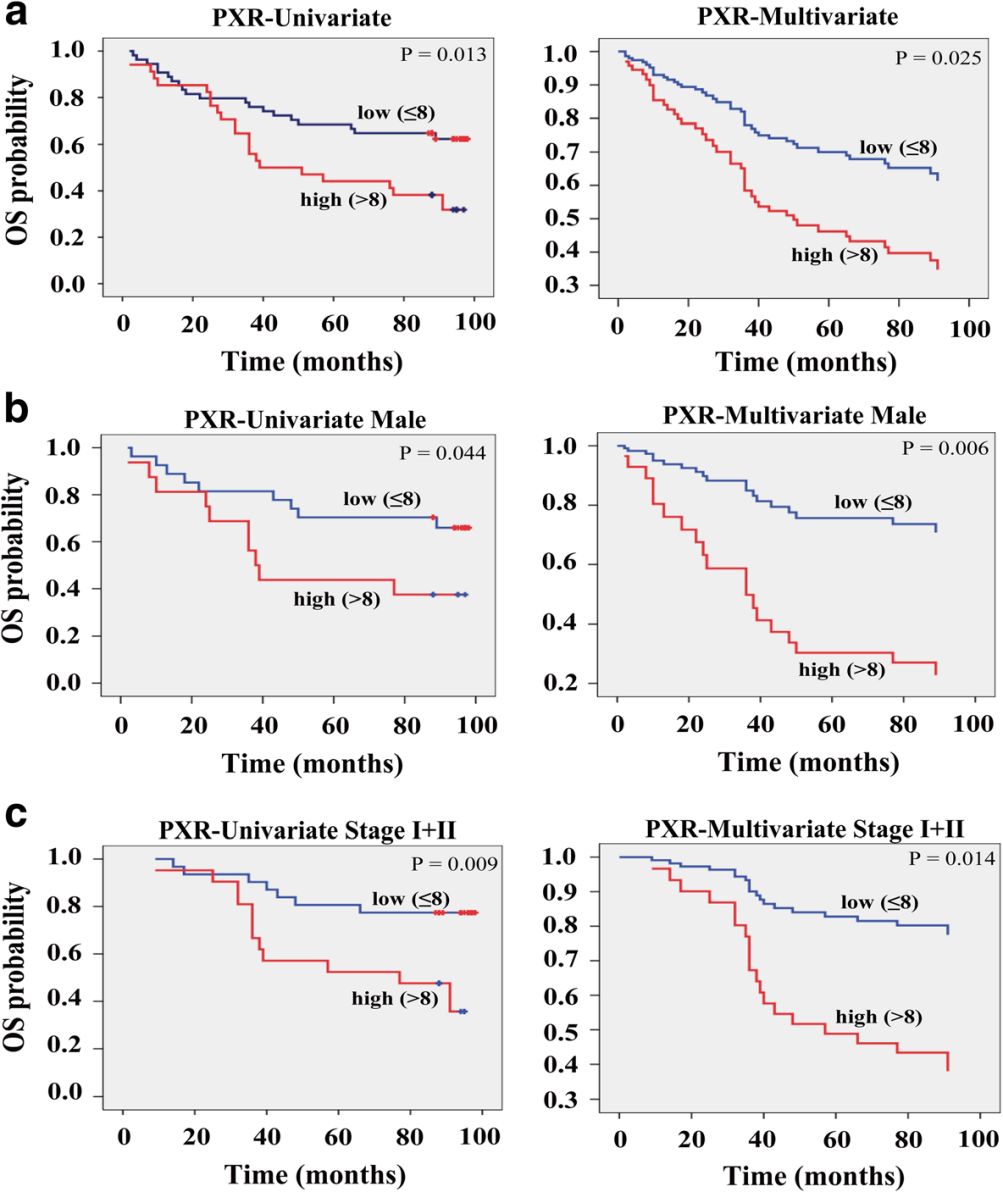
PXR expression is significantly associated with poor overall survival in colorectal cancer patients. **a** Kaplan-Meier (univariate) and Cox regression (multivariate) survival analysis of PXR expression in 93 colorectal cancer patients. PXR expression is associated with poor OS and an independent prognostic factor in colorectal cancer patients. Low, staining weak (≤8); High, staining strong (>8). **b** Kaplan-Meier and Cox regression survival analysis of PXR expression in 47 male colorectal cancer patients. **c** Kaplan-Meier and Cox regression survival analysis of PXR expression in 56 stage I + II colorectal cancer patients

To investigate the correlation between PXR expression and survival of CRC patients, we next examined the contribution of PXR expression to patient OS. Kaplan-Meier survival analysis revealed a poorer OS in cancer patients characterized with high PXR expression compared with patients with low PXR expression (*P* = 0.013; Fig. 7a). To avoid bias, PXR expression and other parameters were examined in a multivariate Cox-regression analysis. PXR expression was found to be an independent prognostic factor [hazard ratio (HR) = 1.463, 95% CI 1.049-2.041, *P* = 0.025], in addition to age (HR = 1.040, 95% CI 1.000-1.081, *P* = 0.049), for patient OS (Fig. 7a, Additional file 1: Table S4 and Additional file 2).

To determine the importance of other clinicopathological features on the correlation between PXR expression and OS of cancer patients, we stratified patients by PXR expression and other clinicopathological features followed by analysis of survival data. The results revealed that PXR expression was significantly associated with OS of male patients (univariate, *p* = 0.044/multivariate, *p* = 0.006) or the patients at clinical stage I + II (univariate, *p* = 0.009/multivariate, *p* = 0.014) (Fig. 7b, c, Additional file 1: Table S5, Table S6 and Additional file 2). However, PXR expression was not statistically correlated with OS of female patients (*p* = 0.134) or the patients at clinical stage III + IV (*p* = 0.247). There are no different impacts about histological grade on the correlation between PXR expression and OS. These findings suggested that PXR was an unfavorable and independent prognostic factor for male or clinical stage I + II CRC patients.

## Discussion

In this study, we investigated the molecular mechanisms of PXR-mediated MRP3 over-expression, which we demonstrate for the first time contributes to chemotherapeutic resistance in CRC. Our data demonstrated that PXR increased the L-OHP efflux capacity, reducing drug concentrations in tumor cells. Functional analysis revealed that PXR blunted L-OHP-mediated inhibition of cellular proliferation and inducing of apoptosis. Our nude mouse experiments showed that PXR reduced the efficacy of L-OHP treatment on tumor growth. TAM analysis revealed increased expression of PXR and MRP3 in cancer tissues and a positive correlation between MRP3 and PXR expression (*P* = 0.0023), which was further confirmed in two independent cohorts from the TCGA database. To investigate the mechanism of PXR-induced L-OHP resistance, MRP3 expression was assessed in cell models that differentially expressed PXR. The results revealed increased MRP3 expression in PXR over-expressing cells. Luciferase reporter and ChIP assays indicated that PXR can bind to the promoter of MRP3 to activate transcription and furthermore, that the PXR-binding sites are located at between -796 to -782bp of the MRP3 promoter. Moreover, we found that PXR expression is an independent predictor for poor prognosis in CRC patients. These results suggest that PXR can transcriptionally activate MRP3 by directly binding to its promoter, increasing drug efflux capacity and resulting in resistance to platinum drugs.

Chemotherapy is the treatment of choice in patients with advanced or metastatic CRC. Chemotherapeutic resistance of tumor cells is crucial problem and a complicated process that involves multiple genes and steps. PXR is a member of the ligand-activated transcription factor superfamily, and its downstream target genes are involved in drug metabolism and transportation [20, 31]. Increasing evidence suggests that high PXR expression is associated with decreased treatment efficacy and increased chemo-resistance against drug-based cancer treatments, including irinotecan, tamoxifen, paclitaxel, doxorubicin and vinblastine [32–39]. In this study, PXR increased the drug efflux capacity and reduced the amount of drug in tumor cells, attenuating the efficacy of L-OHP. PXR expression significantly decreased L-OHP.-mediated tumour cell growth inhibition and inducing of apoptosis. In our nude mouse experiments, PXR decreased L-OHP-mediated suppression of tumor growth. These results demonstrate that PXR enhances the resistance of CRC cells to the chemotherapeutic agent L-OHP.

Previous studies have indicated that PXR regulates a number of genes associated with drug resistance, such as cytochrome P450, multidrug resistance 1 and multidrug resistance‑associated protein 2 [20, 31, 40]. Mounting evidence has shown that PXR is a master regulator of chemotherapeutic drug resistance in cancer treatment. However, the specific mechanism underlying this resistance remains unclear. MRP3 (also called ABCC3) belongs to the ABCC subfamily, which consists of 13 members in mammals and has been divided into the following 3 classes: the multi-drug resistance proteins (MRPs), the sulfonylurea receptors and the cystic fibrosis transmembrane conductance regulator (CFTR/ABCC7). MRP3 has not been extensively studied and its function and regulation remain to be completely established. Moreover, MRP3 is known to transport various bile salts and clinical drugs [41–44]. In this study, our results revealed a positive correlation between MRP3 and PXR expression (*P* = 0.0023), and in three independent cohort, we found increased MRP3 expression in samples expressing high levels of exogenous and endogenous PXR. As PXR is a transcription factor, we used luciferase reporter assays to assess whether PXR binds to the MRP3 promoter to activate its transcription. Our results revealed that PXR can directly bind to the MRP3 promoter to activate its transcription. To determine the precise PXR-binding site within the MRP3 promoter, we constructed luciferase constructs containing different regions of the MRP3 promoter. Luciferase reporter and ChIP assays determined that the PXR-binding sites are located between -884 to -620bp of the MRP3 promoter. Further studies reveal that the key binding sites are “---------**AGGGA**-” (-787 to -783bp) between -884 to -620bp of the MRP3 promoter. These data showed that PXR activates MRP3 transcription by directly binding to its promoter, which increases the drug efflux capacity and results in resistance to platinum drugs. Unfortunately, the crucial sites within the PXR to bind MRP3 promoter are still unknown, and further studies employing site-directed mutagenesis of putative binding sites in PXR are required to clarify this issue. In addition, we also tested other known PXR target genes, and found that the MRP5, MDR1 and OATP-A expression were slightly up-regulated in the PXR over-expression cells (Additional file 1: Figure S1). Although the other target genes also appeared increasing, we still think that PXR increases the drug efflux capacity at least in part by directly activating MRP3 transcription according to our data of function, mechanism and restoring experiments.

To further determine the importance of PXR in CRC, a correlation analysis between PXR expression and the OS of patients was performed. Kaplan-Meier and Cox-regression analysis revealed significantly reduced OS of patients with high PXR expression. Further studies demonstrate that PXR is an unfavorable and independent prognostic factor for male or clinical stage I + II CRC patients. These results indicate that PXR is a potential biomarker for predicting outcome in CRC patients.

## Conclusion

**Fig. 8:**
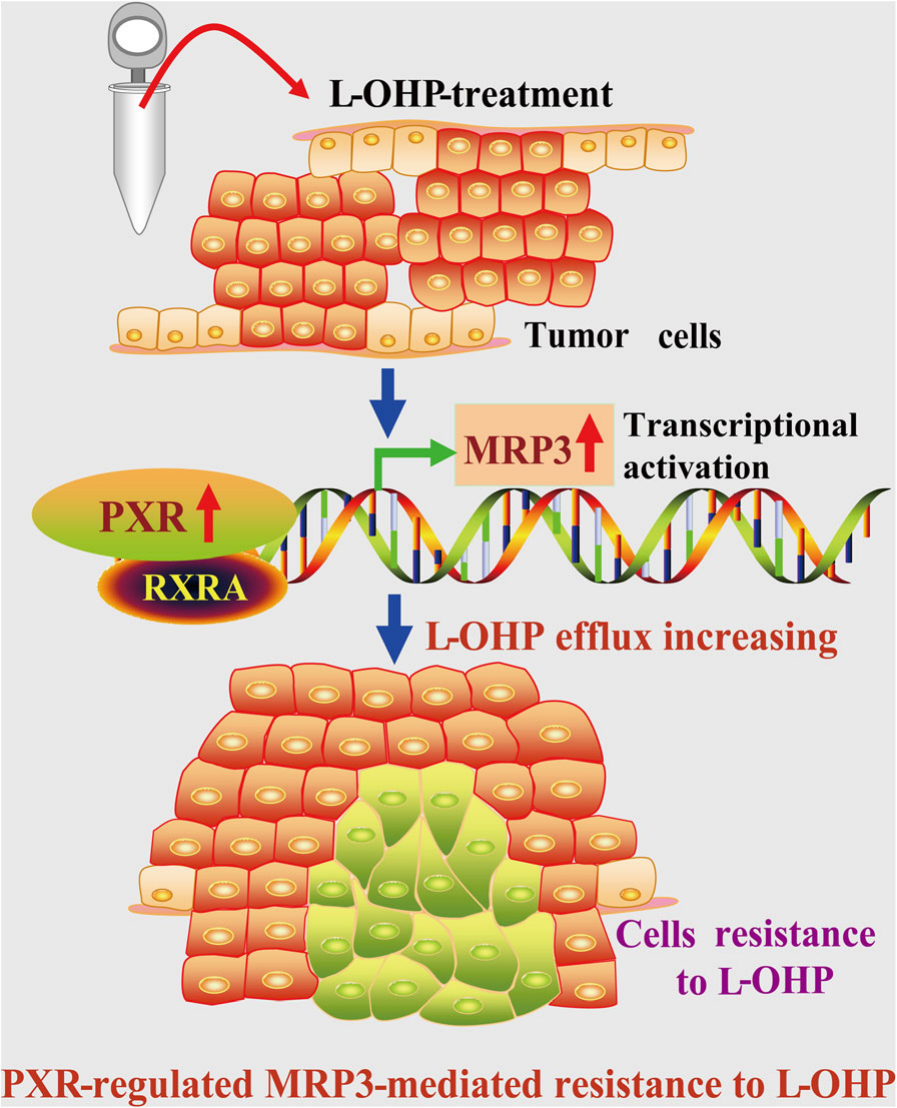
A schematic diagram for the mechanisms underlying PXR-regulated MRP3-mediated resistance. PXR enhances the resistance of tumor cells to the chemotherapeutic agent L-OHP via direct transcriptional activating MRP3 expression, increasing the L-OHP efflux capacity of tumor cells in CRC

In conclusion, PXR enhances the resistance of tumor cells to the chemotherapeutic agent L-OHP via direct transcriptional activating MRP3 expression, increasing the L-OHP efflux capacity of CRC cells (Fig. 8). However, whether this mechanism is generally applicable to all chemotherapeutic drugs, to only specific platinum-based drugs, or only to L-OHP requires further investigation.

## Additional files

Additional file 1: The primers, clone number, correlation of expression with clinicopathological features, prognostic factors and expression of PXR targets in this study. (DOC 289 kb)

Additional file 2: Database of PXR IHC and clinical characteristics of colorectal cancer patients. (XLSX 20 kb)

## Abbreviations

ChIP: Chromatin-immunoprecipitation
CRC: Colorectal cancer
IHC: Immunohistochemical
L-OHP: Oxaliplatin
MRP3: Multidrug resistance-related protein 3
OS: Overall survival
PXR: Pregnane X receptor
s.d.: Standard deviation
TCGA: The Cancer Genome Atlas
TMA: Tissue microarray
WB: Western blot
