# Supplementary material for: Pregnane X receptor is associated with unfavorable survival and induces chemotherapeutic resistance by transcriptional activating multidrug resistance-related protein 3 in colorectal cancer
Source: Mol Cancer. 2017 Mar 29;16:71. doi: 10.1186/s12943-017-0641-8 (PMC5372326; doi:10.1186/s12943-017-0641-8)
Supplement: Supplementary file 1 — The primers, clone number, correlation of expression with clinicopathological features, prognostic factors and expression of PXR targets in this study. (DOC 289 kb) [file 12943_2017_641_MOESM1_ESM.doc]

**Pregnane X receptor is associated with unfavorable survival and induces chemotherapeutic resistance by transcriptional activating multidrug resistance-related protein 3 in colorectal cancer**

Yan Dong^1^, Zhe Wang^1^, Gan-feng Xie^1^, Chong Li^1^, Wen-wei Zuo^1^, Gang Meng^2^, Cheng-ping Xu^2^, Jian-jun Li^1^, ^*^.

^1^ Department of Oncology, Southwest Hospital, Third Military Medical University, Chongqing, China; ^2^ Department of Pathology, Southwest Hospital, Third Military Medical University, Chongqing, China

*Corresponding author

Jian-jun Li

No. 29, Gaotanyan Street, Shapingba District,

Chongqing 400038, PR China

Tel: +86 23 68765193

Fax: +86 23 65425219

E-mail: [jianjunli@tmmu.edu.cn](mailto:jianjunli@tmmu.edu.cn)

**Supplementary tables**

**Tables**

**Table S1 Primer sequences used in this study**

| Primer | Sequence | Purpose |
| --- | --- | --- |
| PXR-F | ATGACAGTCACCAGGACTCAC | Expression analysis (RT-PCR) |
| PXR-R | TGGGTCTCACCTCCAGGTTT |
| MRP3-F | CTGTGCACACAGAAAACCCG |
| MRP3-R | GGACACCCAGGACCATCTTG |
| MRP3-pF | AGGACGAGGACGAGGACACT | ChIP-PCR Analysis |
| MRP3-pR | GAATGGAAAGGGTAGGCAAAGC |
| ACTIN-F | GAGCTACGAGCTGCCTGACGG | Internal control  (RT -PCR) |
| ACTIN-R | CCTAGAAGCATTTGCGGTGG |

**Table S2 The number of clones for colony formation assays**

| Sample | Vector-L-OHP | PXR-L-OHP | P value |
| --- | --- | --- | --- |
| 1 | 76 | 126 |  |
| 2 | 87 | 131 |  |
| 3 | 65 | 119 |  |
| 4 | 48 | 107 |  |
| 5 | 59 | 113 |  |
| **Average** | **67** | **119.2** | **0.0002** |

**Table S3 Correlation of PXR expression with clinicopathological features in human colorectal cancer** patients (n=93)

| PXR expression  Clinical Feature Total High (n=35) Low (n=58) P value | | | | |
| --- | --- | --- | --- | --- |
| Age (years)  <66  ≥66 | 48  45 | 12  23 | 36  22 | **0.011** |
| Sex  Male  Female | 47  46 | 17  18 | 30  28 | 0.832 |
| Histological grade  1  2  3 | 21  65  7 | 8  26  1 | 13  39  6 | 0.410 |
| Lymph node status  Negative  Positive | 57  36 | 23  12 | 34  24 | 0.519 |
| Tumour size  ≤5cm  >5cm | 58  33 | 23  10 | 35  23 | 0.497 |
| Clinical stage  I+II  III+IV | 56  37 | 22  13 | 34  24 | 0.827 |

**Table S4 Multivariate analysis of different prognostic factors in colorectal cancer patients**

| Variable | HR (95% CI) | P value |
| --- | --- | --- |
| Age | 1.040 (1.000-1.081) | **0.049** |
| Sex | 0.939 (0.491-1.799) | 0.851 |
| Histological grade | 1.760 (0.903-3.431) | 0.097 |
| Tumour size | 0.980 (0.800-1.201) | 0.844 |
| Lymph node No | 1.281 (0.958-1.714) | 0.095 |
| Clinical stage | 1.265 (0.577-2.773) | 0.558 |
| PXR expression | 1.463(1.049-2.041) | **0.025** |

**Table S5 Multivariate analysis of different prognostic factors in male colorectal cancer patients**

| Variable | HR (95% CI) | P value |
| --- | --- | --- |
| Age | 1.039 (0.982-1.099) | 0.180 |
| Histological grade | 2.917 (0.769-11.061) | 0.116 |
| Tumour size | 1.039 (0.731-1.477) | 0.832 |
| Lymph node No | 1.151 (0.652-2.032) | 0.627 |
| Clinical stage | 1.729 (0.476-6.278) | 0.405 |
| PXR expression | 2.067 (1.227-3.483) | **0.006** |

**Table S6 Multivariate analysis of different prognostic factors in stage I+II colorectal cancer patients**

| Variable | HR (95% CI) | P value |
| --- | --- | --- |
| Age | 0.977 (0.909-1.409) | 0.514 |
| Sex | 1.114 (0.418-2.969) | 0.829 |
| Histological grade | 1.190 (0.444-3.192) | 0.729 |
| Tumour size | 0.852 (0.632-1.146) | 0.293 |
| PXR expression | 1.947 (1.144-3.313) | **0.014** |

**Supplementary figure and figure legend**

**Figure S1**


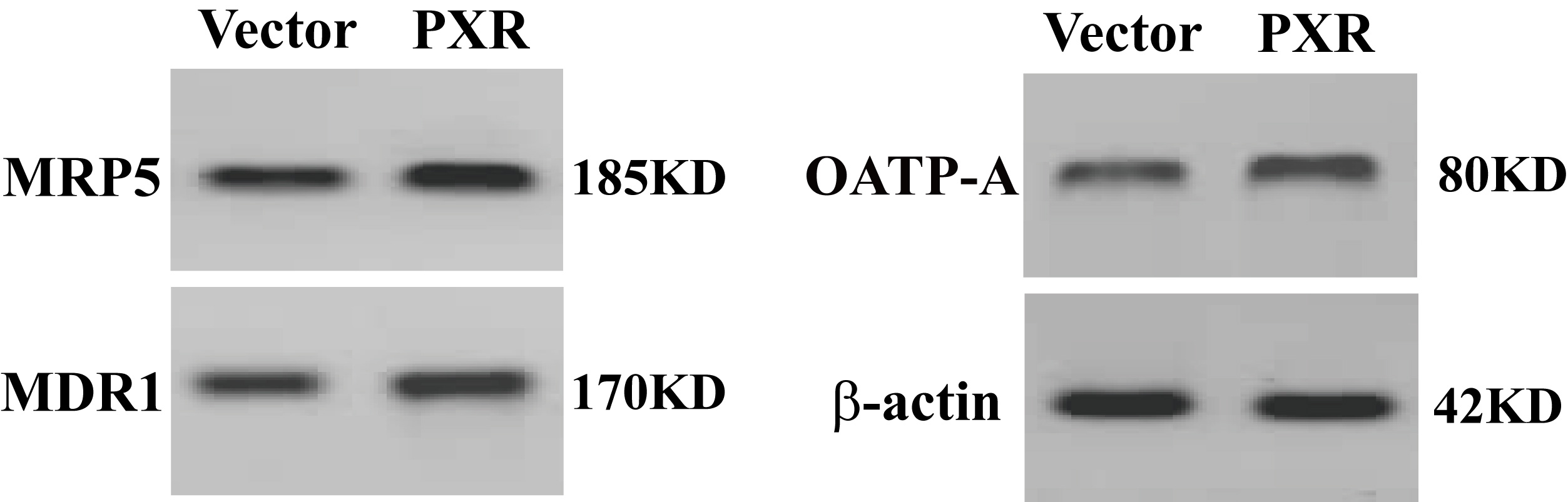


**Figure S1** **The expression of PXR target genes in PXR and empty vector control transfectants by WB.** β-actin was used as an internal control. The primary antibodies are MRP5 mouse monoclonal antibody (1:1,000, Santa Cruz Biotechnology, sc-376965), MDR1 mouse monoclonal antibody (1:1,000, Santa Cruz Biotechnology, sc-55510) and OATP-A mouse monoclonal antibody (1:800; Santa Cruz Biotechnology, sc-365007).
